# A specific cadherin phenotype may characterise the disseminating yet non-metastatic behaviour of pseudomyxoma peritonei

**DOI:** 10.1038/sj.bjc.6603398

**Published:** 2006-10-10

**Authors:** R Bibi, N Pranesh, M P Saunders, M S Wilson, S T O'Dwyer, P L Stern, A G Renehan

**Affiliations:** 1 Cancer Research UK Immunology Group, Paterson Institute for Cancer Research, Manchester, UK; 2 Department of Surgery, Christie Hospital NHS Trust, Manchester, UK; 3 Department of Clinical Oncology, Christie Hospital NHS Trust, Manchester, UK

**Keywords:** pseudomyxoma peritonei, cytokeratins, cadherin, vimentin

## Abstract

Pseudomyxoma peritonei (PMP) is a rare neoplasm of mainly appendiceal origin, characterised by excess intra-abdominal mucin production leading to high morbidity and mortality. While histological features are frequently indolent, this tumour disseminates aggressively throughout the abdominal cavity, yet seldom metastasises. This study determined the expression of several markers of colorectal differentiation (carcinoembryonic antigen (CEA), cytokeratins (CK20 and CK7), epithelial membrane antigen), mucin production (MUC-2, interleukin-9 (IL-9), IL-9 receptor (IL-9R*α*)), and cell adhesion (N- and E-cadherin, vimentin) in PMP tissue (*n*=26) compared with expressions in normal colonic mucosa (*n*=19) and colorectal adenocarcinoma (*n*=26). Expressions of CEA and cytokeratins were similar for PMP as those in colorectal adenocarcinomas with the exception that the CK20−/CK7− pattern was rare in PMP (Fisher's exact test: *P*=0.001). Similarly, expressions of mucin-related proteins were comparable for adenocarcinoma and PMP, with the exception that IL-9 expression was uncommon in adenocarcinoma (*P*=0.009). Pseudomyxoma peritonei demonstrated a specific pattern of adhesion-related protein expressions of increased N-cadherin, reduced E-cadherin, and increased vimentin (*P*=0.004), a phenotype suggesting a possible epithelial–mesenchymal transition state. Primary PMP cell cultures were successfully maintained and demonstrated marker expressions similar to those seen in *in vivo* tissues. These early characterisation studies demonstrate similarities between PMP and colorectal adenocarcinoma, but also reveal a specific cadherin phenotype that may characterise the distinct non-metastasising behaviour of PMP, and form the basis for future mechanistic and therapy-targeting research.

Pseudomyxoma peritonei (PMP) is a rare neoplasm (one per million population per year) defined by disseminated peritoneal mucinous tumour deposition and progressive accumulation of mucinous ascites (Hinson and Ambrose, 1998; Esquivel and Sugarbaker, 2000; Bryant *et al*, 2005). At a clinical level, PMP is characterised by a tendency to progress despite surgery, with ultimate high mortality. There are three pathological types: (i) disseminated peritoneal adenomucinosis (DPAM), the commonest; (ii) peritoneal mucinous carcinomatosis with intermediate and/or discordant features (PMCA-I/D); and (iii) peritoneal mucinous carcinomatosis (PMCA), with respective 5-year age-adjusted survival rates of 84, 38, and 7% (Ronnett *et al*, 1995b).

Originally thought to be of ovarian origin, it is now widely accepted that the appendix is the source of neoplasia in PMP (Hinson and Ambrose, 1998; Esquivel and Sugarbaker, 2000). While the appendiceal lesion often maintains indolent histological features, the disease may disseminate aggressively throughout the abdominal cavity, but notably seldom metastasises. The accumulation of extracellular mucin is central to the pathogenesis of the disease and contributes to its morbidity and mortality. However, because of its rarity, the molecular investigation of this disease has been scarce (Mohamed *et al*, 2004).

In the United Kingdom, since 2001, the Department of Health have centralised the management of PMP to two centres (Sugarbaker, 2006), offering opportunities for the collection of tissue samples to undertake early characterisation studies.

This study aimed to investigate markers that might be relevant to the pathology and progression in PMP. The expression patterns of markers such as the carcinoembryonic antigen (CEA), cytokeratins (CK20 and CK7), and epithelial membrane antigen (EMA) were primarily determined as these reflect the origin of disease. To address the specific feature of excess extracellular mucin production, we determined the differential expression of MUC-2, a mucin with gel-forming physicochemical properties similar to those exhibited by PMP (Kim and Gum, 1995), as well as interleukin-9 (IL-9) and its receptor (IL-9R*α*), as IL-9 has been implicated in mucin-producing conditions such as cystic fibrosis (Hauber *et al*, 2003) and asthma (Louahed *et al*, 2000). To evaluation the features of PMP dissemination, we determined the expression of molecules involved in cell adhesion and invasion, specifically N- and E-cadherin, and vimentin, as these are important in colorectal (Dorudi *et al*, 1993; Rosivatz *et al*, 2004) and other carcinomas (Bringuier *et al*, 1993; Islam *et al*, 1996; Hazan *et al*, 1997; Rubin *et al*, 2001). In particular, we sought to determine the phenotype of N-cadherin+/E-cadherin−/vimentin+, as this has been implicated in epithelial–mesenchymal transition (EMT) (Tran *et al*, 1999), a process important in the progression of neoplasia towards dedifferentiation and more ‘malignant’ states (Thiery, 2002). To these ends, studies were undertaken in *in vivo* tissues (normal colon, colorectal cancer, PMP) and *in vitro* primary derived PMP cell lines.

## MATERIALS AND METHODS

### Patients

Following local ethical approval (LREC-02/051), tissues were collected from patients undergoing surgery at the Christie Hospital NHS Trust, Manchester, UK, between 2001 and 2004. There were three tissue groups – normal colonic mucosa (*n*=19), colorectal adenocarcinoma (*n*=26), and PMP (*n*=26) – with demographic and clinicopathological characteristics as listed in Table 1.

Formalin-fixed, paraffin-embedded tissue blocks were prepared for immunohistochemistry at the Paterson Institute for Cancer Research (PICR). Samples of histologically normal colonic mucosa were taken >10 cm from tumours in patients undergoing colorectal resection, and orientated to obtain a maximum number of longitudinal crypts before final embedment (Renehan *et al*, 2002). All colorectal adenocarcinoma samples were from patients undergoing primary surgical resection without preoperative chemotherapy and/or radiation therapy, and without a histological diagnosis of mucinous adenocarcinoma. Fifteen of the adenocarcinoma group formed paired samples with the normal colonic mucosa group. For PMP tissue, wherever possible, the primary appendiceal neoplasm was used (*n*=14) but where not available (due to prior appendicectomy), cellular elements of PMP were judged on multiple haematoxylin- and eosin-stained sections from other sites to identify the most representative block.

### Immunohistochemistry

Antibodies were selected to examine three broad groups of markers – tissue differentiation, mucin production, and cell adhesion – and their suitability for formalin-fixed, paraffin-embedded tissue as shown in Table 2.

Five-micrometre-thick sections were cut and deparaffinised in graded alcohols and xylene. The sections were then subjected to immunostaining, applying a standard avidin–biotin–peroxidase method by hand as described elsewhere in experiments from the PICR (Starzynska *et al*, 1997; Wilson *et al*, 2000). Before Ki-67 and vimentin immunostaining, antigen retrieval was carried out by boiling the sections in citrate buffer (pH 6.0) for 2 × 10 min using a microwave oven. For all antibodies, staining was completed with 2–3 min incubation with diaminobenzidene using the substrate-chromogen solution supplied in the EnVision™ HRP kit (DAKO, Cambridge, UK). Slides were counterstained with haematoxylin and reviewed. Appropriate positive and negative controls were used throughout (webappendix).

### Scoring of immunostaining

Immunostaining was scored semiquantitatively using the immunohistochemical score (IHS) method of assessment, a method shown to have a high level of inter-laboratory correlation (Rhodes *et al*, 2000) and to approximate data generated from image analysis-based scoring systems (Remmele and Schicketanz, 1993). For each section, the IHS was the addition of the staining intensity (zero, no staining; 1, weak; 2, moderate; 3, strong) and percentage of positive tumour cells (0, none; 1 for 1–25%; 2 for 26–50%; 3 for 51–75%; 4 for 76–100%) with a range of 0–7 for each specimen. An IHS value ⩾2 was considered positive. Because of the sparse epithelial nature of PMP, a learning set of six samples was used to explore histopathological features and their associated immunostaining before the main study. In addition, owing to the high mucin component, epithelial cells were identified by immunostaining with EMA before scoring. For IHS scoring, the intra-observer agreement was 73% (based on 40 specimens stained for E-cadherin; *κ*=0.628, s.e.=0.085) and inter-observer agreement ranged from 66 to 86% (based on two independent investigators, RB and NP, scoring all specimens stained for IL9Rα: *κ*=0.571, s.e.=0.059, and N-cadherin: *κ*=0.776, s.e.=0.072).

### Establishment of PMP cell lines

Pseudomyxoma peritonei cell cultures were established from intra-abdominal diseased tissue, paired with controls cultured from skin obtained from tissue excised around the laparotomy scar. Cellular elements were finely minced using a sterile scalpel and pipetted into small aggregates. These were seeded either into six-well plates containing minimal medium (DMEM; 10% FCS; L-glutamine (2 mM); penicillin–streptomycin (0.1 mg ml^−1^)) or ALC-4 serum-containing medium (RPMI 1640; 10% FCS; L-glutamine (2 mM); penicillin–streptomycin (0.1 mg ml^−1^); insulin (0.02 mg ml^−1^); transferrin (0.01 mg ml^−1^); sodium selenite (25 nM); hydrocortisone (50 nM); epidermal growth factor (1 ng ml^−1^); ethanolamine (0.01 mM); phosphorylethanolamine (0.01 mM); tri-iodothyronine (0.1 nM); HEPES (10 mM); sodium pyruvate (0.5 mM)), a fully defined medium formulated for the selective growth of human lung adenocarcinoma cells (Brower *et al*, 1986). The cells were subsequently cultured at 37°C in 5% CO_2_ changing half of the medium every 2–3 days. The cultures were first split after 3–4 weeks, and thereafter passaged at this time interval at 1 : 2 ratios.

### Immunocytochemical analysis

Paraffin-embedded blocks were prepared from PMP cell cultures taken from passages 6 and 10, which were washed with PBS and fixed with formalin overnight. In addition, cells were cultured on eight-well chamber slides from passages 6 and 10 until cells were 80% confluent, washed with PBS, and fixed with 4% paraformaldehyde for 15 min. The DAB–peroxidase system (DAKO, Cambridge, UK) was used for immunocytostaining using the same panel of antibodies as described above.

### Statistical analysis

SPSS version 14.0 (Superior Performing Software Systems, Chicago, IL, USA) statistical program was used for statistical analyses. Categorical data, for example, proportions of positive tumours per group, were compared using the *χ* ^2^ and Fisher's exact tests as appropriate. The IHS scores were ordinal and were presented as turnip plots using the turnip command in STATA version 8.2 (College Station, TX, USA), and compared using the Mann–Whitney *U*-test. Paired comparisons were performed using the Wilcoxon signed rank test. As there were multiple comparisons, only *P*-values <0.01 were considered to be statistically significant.

## RESULTS

### Immunopositivity for *in vivo* tissue

The immunopositivities for normal colonic mucosa, colorectal adenocarcinoma, and PMP tissues for markers of tissue differentiation and mucin production are shown in Table 3. Expressions of CEA and CK20, with absent CK7, was universal in well-orientated normal colonic mucosa. Expression of CEA was also universal among adenocarcinomas and present in all but one PMP specimen. The patterns of combined CK20 and CK7 were similar for adenocarcinomas and PMP, with the exception that the CK20−/CK7− pattern was not seen in PMP tissue series (Fisher's exact test: *P*=0.001).

At least some MUC-2 immunopositivity was noted in all three tissue groups, reflecting the presence of goblet cells. Similarly, the IL9R*α* was almost universally present in all three tissue groups. However, IL9 expression was relatively uncommon in adenocarcinoma compared with PMP tissue (*P*=0.009).

Expression of N-cadherin was almost absent in normal colonic mucosa, increased in adenocarcinoma (46%), and present in over two-thirds (68%) of PMP tissues (*P*<0.001). There was a suggestion that expression was increased in advanced adenocarcinomas compared with early tumours, but sample numbers were small. By contrast, expression of E-cadherin expression was universal in normal and adenocarcinoma tissues, but reduced in PMP tissue (80%). Expression of vimentin was absent in normal and adenocarcinoma tissues, but present in over a third (36%) of PMP tissues (*P*=0.004).

### Immunohistochemical scores

**Figure 1:**
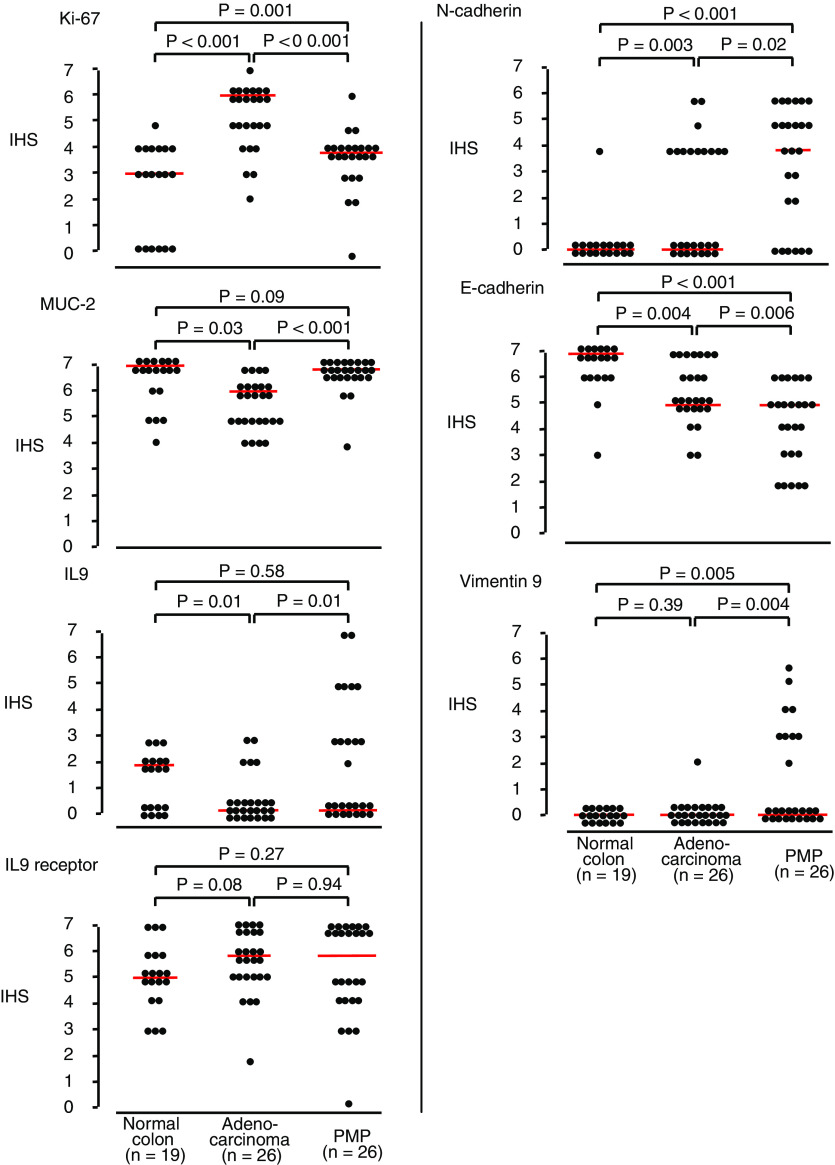
IHS scores for expression of Ki-67, MUC-2, IL9, IL9 receptor, N-cadherin, E-cadherin, and Vimentin in normal colonic mucosa, adenocarcinoma and PMP tissues. The intensity of staining was evaluated using the IHS method of assessment. The intensity of staining was recorded as follows: 0, negative (no staining); 1, weak; 2, moderate; 3, strong (strikingly positive). The proportion of cells in the PMP component showing positive staining was recorded as follows: 0, none; 1, approximately 1–25%; 2, 26–50%; 3, 51–75%; 4, 76–100%. The score for intensity was added to the score for proportion, giving the IHS score, with a range of 0–7 for each specimen. Plots were generated using the turnip command in STATA (version 7). Comparisons of IHS scores used Mann–Whitney *U* independent tests and *P*-values (two-sided) expressed above each comparison in the figure. Among the samples of normal colonic mucosa and adenocarcinoma, there were 15 paired samples and were compared using the Wilcoxon signed rank test. Where independent tests were statistically significant, the paired tests were as follows: N-cadherin, *P*=0.05; E-cadherin, *P*=0.004; Ki-67, *P*=0.001.

To further explore the relative differences between normal colonic mucosa, colorectal adenocarcinoma, and PMP, IHS medians were compared (Figure 1). Not unexpectedly, Ki-67 IHS median was significantly increased in adenocarcinomas (*P*<0.001) compared with normal colonic mucosa; in turn, PMP IHS median was similar to that for normal mucosa. Consistent with the clinicopathological feature of excess mucin production, the IHS scoring revealed that MUC-2 expression was increased in PMP tissue compared with adenocarcinoma (*P*<0.001).

For the cell adhesion-related proteins, a specific phenotype emerged. N-cadherin expression was significantly raised in adenocarcinoma specimens compared with normal mucosa (*P*=0.003), and was similarly raised in PMP tissue (*P*<0.001). Although E-cadherin immunostaining was broadly positive in all three groups, the IHS scoring demonstrated a stepwise reduction in expression from normal mucosa to adenocarcinoma (*P*=0.004), and from adenocarcinoma to PMP (*P*=0.006). The IHS scoring confirmed that vimentin expression was significantly increased in PMP tissue (*P*=0.004).

### Pseudomyxoma peritonei *in vitro* cell lines

**Figure 2:**
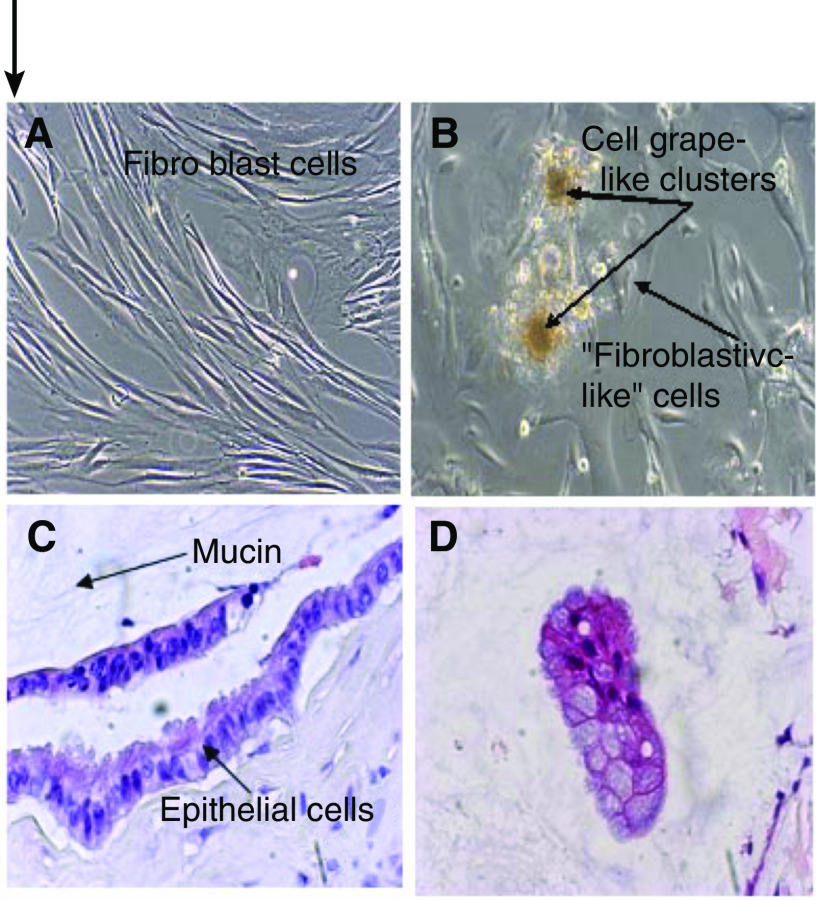
Primary cultures of diseased PMP tissue and paired abdominal skin. Phase-contrast images of the cell cultures grown in the enriched ALC-4 medium. (**A**) Fibroblast cells grown in skin. (**B**) PMP culture at early passage 2 showing the two cell types – ‘fibroblastic’ cell type and grape-like clusters. One of the PMCA (PMP12) cultures grew well until passage 12, thereafter we observed that the grape-like cell clusters were lost and only fibroblastic-like cells grew to passage 15 and then senesced; the other (PMP17) continued to grow well and maintained the two cell types. PMP15 and PMP16 cultures were established from DPAMs and became senescent at passages 8 and 12, respectively. (**C**) Haematoxylin- and eosin-stained section demonstrating typical features of adenomucinosis ‘epithelium’ surrounded by a pool of mucin. (**D**) Cell pellet preparation of primary PMP cell culture from the same patient as (**C**), illustrating the same phenotype as the *in vivo* specimen ( × 100 magnification).

Primary cultures were successfully established in PMP samples from 17 patients, grown for at least three passages, and recoverable from cryopreservation. Four lines were maintained in culture for over 12 months. Early passages (from 1 to 10) were characterised by two cell types: an adherent layer of ‘fibroblastic-like cells’ and a weakly adherent grape-like cluster cell colony. When the latter were isolated, selected, and seeded, the two cell types were reformed. By contrast, where skin cultures were established, all 17 showed a uniform fibroblastic morphology with no grape-like cluster colonies (Figure 2A and B).

Mucin was demonstrated by Alcian blue–periodic acid–Schiff staining (pH 2.5) in both *in vivo* and *in vitro* settings, suggesting that the cultured PMP cells produce mucin. The labelling for mucin in PMP cultures was associated with both the grape-like cell colonies and most of the fibroblastic-like cells. A cell pellet preparation from a PMP17 line at passage 5 stained with haematoxylin and eosin revealed the typical goblet cell morphology, which compares well with the original PMP ‘tumour’ section (Figure 2C and D).

**Figure 3:**
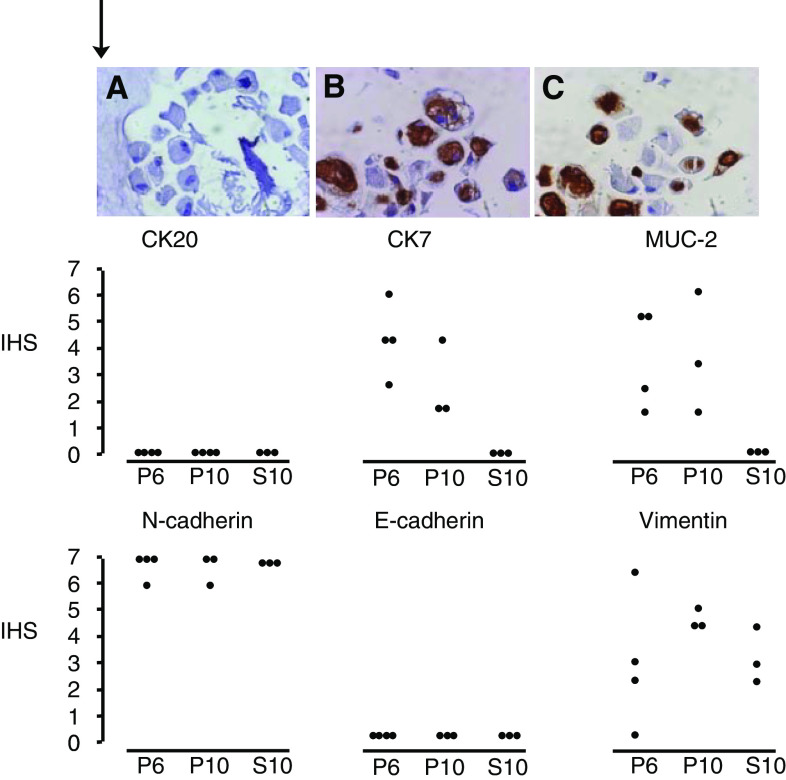
Paraffin-embedded sections of cell pellet preparations from a primary PMP cell culture and immunocytochemical analyses. (**A**) Isotype control IgG1 showing no immunostaining. (**B**) Positive MUC-2 staining of cultured PMP cells. (**C**) Positive CK7 staining of cultured PMP cells ( × 100 magnification). Plots for immunocytochemical staining for CK20, CK7, MUC-2 (middle panel) and N-cadherin, E-cadherin, vimentin expression (lower panel) in four PMP lines stained at 6 weeks (P6) and 10 weeks (P10) compared with paired skin lines at 10 weeks (S10). IHS: immunohistochemical score.

The cultured PMP cells did not express CK20, CEA, or E-cadherin (*n*=4), even though the primary samples *in vivo* were positive for these markers. However, the cultured PMP cells from passages 6 and 10 showed heterogeneous expression of CK7 and MUC-2, whereas the matched autologous scar wound fibroblast cells were negative for both markers (Figure 3). The MUC2 expression in the cultured PMP cells demonstrates the mucin-producing ability of these cells. All four PMP cultures and their matched wound fibroblast cells expressed N-cadherin and vimentin with the exception of PMP15 culture, which was negative for vimentin.

## DISCUSSION

Since its original description by Rotikansky in 1842 (Weaver, 1937), much of the study of PMP has focused on debates of origin (Ronnett *et al*, 1995a) and degree of malignancy (Seidman *et al*, 1993; Misdraji *et al*, 2003), and more recently, on the merits of aggressive cytoreductive surgery (Bryant *et al*, 2005; Sugarbaker, 2006). Because of its rarity, there have been few studies exploring its molecular characteristics. This study determined the expression of four marker groups – differentiation, cell proliferation, mucin secretion, and cell adhesion – using normal colonic mucosa and colorectal adenocarcinomas as comparators. The most striking finding was the presence of specific cadherin and vimentin phenotype. Specifically, normal colonic epithelium is characterised by E-cadherin expression but lack of N-cadherin and vimentin expression; adenocarcinoma is characterised by increased N-cadherin expression in a stepwise manner with advancing stage (Rosivatz *et al*, 2004), but minimal vimentin expression; and PMP has high expression of N-cadherin and reduced E-cadherin expression. This cadherin phenotype is suggestive of an EMT state, a process important in the progression of neoplasia towards dedifferentiation and more ‘malignant’ states. Additionally, the epithelium of a third of PMP tumours expressed vimentin, usually a marker of mesenchymal cells such as fibroblasts, but also interpreted as the hallmark of EMT as cell–cell and cell–extracellular matrix interactions are altered with switching of epithelial cell to mesenchymal phenotypes (Chen *et al*, 2005). These observations may explain one of PMP's unique behaviours – its ability to disseminate widely throughout the abdominal cavity, often aggressively, but seldom metastasise either to nodal or distant sites.

There are two notable limitations in the interpretation of these findings. Firstly, the sample sizes were relatively small, prohibiting subgroup analyses. Previous studies have shown correlations of increased N-cadherin/reduced E-cadherin expression with advancing stage (Rosivatz *et al*, 2004) and tumour grade (Bendardaf *et al*, 2005) in colorectal adenocarcinomas. The former was partly observed in this series, and is being addressed in ongoing studies. Secondly, this study did not measure mRNA expression. However, studies have shown that the expression of cadherin proteins in colorectal tissue is closely correlated with respective gene expression (Dorudi *et al*, 1993).

Advantages of the study include a PMP dedicated research programme, within a single treatment centre, affording a well-characterised clinicopathological data set with uniformity of staging and pathological reporting. Additionally, as a forerunner to the immunohistochemical experiments, a learning set of PMP samples was used to explore histopathological features and their associated immunostaining. This is crucial, as the cellular components on PMP may be sparse and many sections may be required to establish a representative sample in a given patient. For the first time, this study established PMP cell lines that were representative of the disease and could be maintained in culture for more than 1 year. The cell lines shared the *in vivo* phenotype expressing MUC-2, CK7+, and vimentin, but in contrast to the prevalent PMP phenotype, did not express CEA or CK20 and only showed weak expression of EMA. The reasons for these differences are not clear. All but one of the PMP specimens expressed CK20 *in vivo*, supporting an intestinal origin, yet failed to express this marker *in vitro*. The distinct regulation of these markers could reflect differences of *in vitro* culture conditions *versus* *in vivo* expression. The PMP cell lines also strongly expressed N-cadherin, and interestingly, demonstrated both fibroblastic and mucin-secreting cell types on phase contrast, reminiscent of the observations of Islam *et al* (1996) for a squamous cell carcinoma-derived cell line that overexpressed N-cadherin. The ability to reproducibly establish and maintain PMP cell cultures will serve as a valuable tool to explore the molecular basis of PMP in future studies.

In addition to cell adhesion molecules, this study also examined other groups of molecules relevant to PMP pathology. Consistent with the theory that the majority of PMP are colorectal in origin, all PMP tissues were CEA positive, and all but one was CK20 positive. Similar to other studies, this study showed that the CK20−/CK7+ pattern is rare in colorectal adenocarcinomas (and then only found in rectal mucosal lesions) (Lagendijk *et al*, 1999; Hernandez *et al*, 2005), and this extends to PMP specimens. The reporting of CK20 positivity is variable in normal colonic epithelium as it is necessary (as in the current study) to have well-orientated mucosal sections to demonstrate staining in cells in the outer differentiated zone of the intestinal crypt (Hernandez *et al*, 2005). As in other studies (O'Connell *et al*, 2002; Mohamed *et al*, 2004), we demonstrated increased expression of MUC-2 in PMP tissue compared with normal mucosa and adenocarcinoma. Interleukin-9, which induces MUC-2 and MUC-5AC gene expression in the lung, and its receptor, IL-9R*α*, were expressed at variable levels in all cases of PMP with increased IL-9 expression in the epithelial PMP cells compared with adenocarcinoma. It is conceivable that the differential IL-9 expression in PMP might contribute to an autocrine loop leading to goblet cell hyperplasia. The availability of cultured PMP cells will facilitate future understanding of the role of IL-9 in mucin production.

Although originating in the appendix, the disease progression of PMP is very different from other neoplasia arising from other hindgut sites. However, the observations of this study have led us to postulate that there are some molecular pathways common to both PMP and colorectal adenocarcinoma. The majority of colorectal adenocarcinomas develop through the adenoma–carcinoma sequence, a multi-stage process of molecular aberrations and mutations in key oncogenes and tumour suppressor genes (Vogelstein *et al*, 1988; Kinzler and Vogelstein, 1998). In common with the majority of colorectal adenocarcinoma, PMP probably arises through an adenoma, specifically an appendiceal adenoma, but disseminates throughout the abdominal cavity, mainly by peritoneal spread, yet seldom metastasises. Like colorectal adenocarcinoma, this process is characterised by increased N-cadherin and decreased E-cadherin expression, although this may be more exaggerated in PMP and is also associated with increased vimentin expression. In some PMP cases, there is perforation of the appendix with potential ‘spillage’ of adenoma cells (Young, 2004), but it is unclear whether this is a necessary step in the dissemination of PMP. It is conceivable that sporadic adenocarcinoma contain a different, perhaps more expanded, molecular profile to that of PMP that affords the ability to metastasise – questions that will form future research studies.

This study forms the basis to develop further targeted research programmes both in *in vivo* and *in vitro* PMP systems. Larger sample sizes are required such that correlations with survival and other outcomes will be feasible. The genetic regulation of the biological hallmarks of this unusual disease is the key research question for future strategies to prevent and/or inhibit the progression of this debilitating and fatal disease.

## Figures and Tables

**Table 1:** Baseline characteristics

	**Normal colonic mucosa**	**Adenocarcinoma**	**PMP^a^**
No. of samples	19	26	26
Median age (range)	70 (32–86)	71 (36–94)	56 (33–84)^b^
male : females	8 : 11	11 : 15	5 : 21
			
*Staging*			
Early (Dukes' A and B)	—	17 (65)	—
Advanced (Dukes' C and D)	—	9 (35)	—
			
*Degree of differentiation*			
Well	—	5 (19)	—
Moderate	—	16 (62)	—
Poorly	—	5 (19)	—
			
*PMP classification*			
DPAM	—	—	20 (77)
PMCA	—	—	6 (23)

Unless otherwise stated, values in parentheses are percentages.

PMP=pseudomyxoma peritonei; RT=radiotherapy; DPAM=disseminated peritoneal adenomucinosis; PMCA=peritoneal mucinous carcinomatosis.

a Treatment of patients with PMP: 10 cytoreductive resections with hyperthermic intra-operative chemotherapy; 16 debulking laparotomies.

b Pseudomyxoma peritonei patients were significantly younger compared with the other groups (Kruskal–Wallis test; *P*=0.004).

**Table 2:** Antibodies used for immunostaining

**Antibody**	**Dilution**	**Antigen**	**Main specificity**	**Source**
Parlam	1 : 2000	CEA	Gastrointestinal tract epithelium	DAKO, Cambridge, UK
Ks 12.8	1 : 100	CK20	Colonic epithelium	DAKO, Cambridge, UK
OVTL 12/30	1 : 80	CK7	Glandular epithelium/rectal epithelium	Abcam, Cambridge, UK
E29	1 : 100	EMA	Epithelial membrane	DAKO, Cambridge, UK
Ccp58	1 : 100	MUC-2	Mucin-secreting cells including goblet cells	Abcam, Cambridge, UK
Polyclonal	1 : 200	IL-9	Mucin-producing epithelium	Biogenesis, Poole, UK
33401	1 : 100	IL-9R*α*	Mucin-producing epithelium	R&D Systems Inc., Minneapolis, MN, USA
MIB-1	1 : 100	Ki-67	Cell cycle except G_0_	DAKO, Cambridge, UK
32	1 : 100	N-cadherin	Inter-cellular adhesion molecules	BD Transduction Laboratory, Franklin Lakes, NJ, USA
NCH-38	1 : 100	E-cadherin	Inter-cellular adhesion molecules	DAKO, Cambridge, UK
Vim 9	1 : 2000	Vimentin	Mesenchymal cells	Abcam, Cambridge, UK

PMP=pseudomyxoma peritonei; CEA=carcinoembryonic antigen; CK=cytokeratin; EMA=epithelial membrane antigen; MUC=mucin-secreting gene product; IL=interleukin.

**Table 3:** Comparison of immunopositivities for markers of tissue differentiation and mucin production

	**No. of cases (%)**						
	**Normal colonic mucosa**		**Adenocarcinoma**	**PMP**		***P* value^a^**	***P* value^b^**
*Baseline CEA/cytokeratin*							
CEA+	19 (100)		26 (100)	25 (96)		NA	1.0
CK20+/CK7+	0		3 (11)	8 (31)		0.01	0.17
CK20+/CK7−	19 (100)		12 (46)	17 (65)		0.006	0.16
CK20−/CK7+	0		1 (4)	1 (4)		1.0	1.0
CK20−/CK7−	0		10 (39)	0		NA	0.001
EMA	19 (100)		25 (100)^c^	24(100)^c^		NA	NA
							
*Mucin-associated proteins*							
MUC-2	19 (100)		26 (100)	26 (100)		NA	NA
IL-9	3 (16)		2 (8)	11 (42)		0.10	0.009
IL-9R*α*	19 (100)		25 (96)	25 (96)		1.0	1.0
							
*Adhesion-related proteins*							
N-cadherin	1 (5)		12 (46)	17 (68)^d^		<0.001	0.11
E-cadherin	19 (100)		26 (100)	20 (80)^d^		0.06	0.02
Vimentin	0		0	8 (36)		0.01	0.004
							

		**Early**	**Advanced**	**DPAM**	**PMCA**		
N-cadherin		5 (29)	7 (78)	12 (63)^d^	5 (83)		
E-cadherin		17 (100)	9 (100)	15 (79)^d^	5 (83)		
Vimentin		0	0	6 (30)	2 (33)		

Values in parentheses are percentages. PMP=pseudomyxoma peritonei; CEA=carcinoembryonic antigen; CK=cytokeratin; EMA=epithelial membrane antigen; MUC=mucin-secreting gene product; IL=interleukin; DPAM=disseminated peritoneal adenomucinosis; PMCA=peritoneal mucinous carcinomatosis.

a Comparison of PMP with normal colonic mucosa. *χ*^2^ test and Fisher's exact tests as appropriate.

b Comparison of PMP with adenocarcinoma. *χ*^2^ test and Fisher's exact tests as appropriate.

c Missing data: one adenocarcinoma; two PMP cases.

d Missing data: one PMP case.
