# Supplementary material for: A specific cadherin phenotype may characterise the disseminating yet non-metastatic behaviour of pseudomyxoma peritonei
Source: Br J Cancer. 2006 Oct 10;95(9):1258–64. doi: 10.1038/sj.bjc.6603398 (PMC2360585; doi:10.1038/sj.bjc.6603398)
Supplement: Supplementary Figure 1 [file 95-6603398x1.doc]

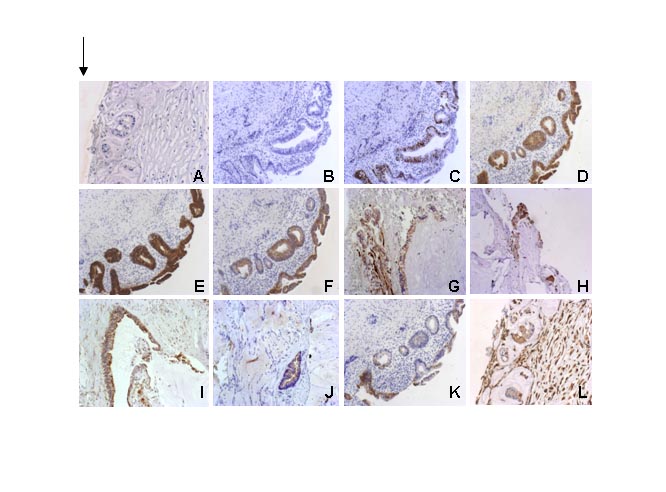


**Webappendix Immunohistochemistry on PMP tissue.**

(A) Isotype control rabbit IgG. (B) Isotype control mouse IgG1. (C) Representative example of Ki-67 experiment showing nuclear staining. (D) EMA staining showing epithelial cell component in PMP. (E) MUC-2 positivity. (F) CK20 positivity. (G) CK7 positivity. (H) Vimentin positivity. (I) IL-9Rα positivity. (J) IL-9 positivity. (K) E-cadherin positivity. (L) N-cadherin positivity including intense positive stromal staining (x100 magnification).
